# Identification of Transforming Hepatitis B Virus S Gene Nonsense Mutations Derived from Freely Replicative Viruses in Hepatocellular Carcinoma

**DOI:** 10.1371/journal.pone.0089753

**Published:** 2014-02-24

**Authors:** Shiu-Feng Huang, Ya-Ting Chen, Wei-Chen Lee, Il-Chi Chang, Yu-Ting Chiu, Yu Chang, Hsiao-Chen Tu, Chiou-Hwa Yuh, Isao Matsuura, Liang-Yu Shih, Ming-Wei Lai, Hong-Dar Isaac Wu, Miin-Fu Chen, Chau-Ting Yeh

**Affiliations:** 1 Institute of Molecular and Genomic Medicine, National Health Research Institutes, Miaoli, Taiwan; 2 Department of Pathology, Chang-Gung Memorial Hospital, Taoyuan, Taiwan; 3 Department of General Surgery, Chang-Gung Memorial Hospital, Chang-Gung University College of Medicine, Taoyuan, Taiwan; 4 Department of Pediatrics, Chang-Gung Memorial Hospital, Chang-Gung University College of Medicine, Taoyuan, Taiwan; 5 Department of Applied Mathematics and Institute of Statistics, National Chung-Hsing University, Taichung, Taiwan; 6 Liver Research Center, Chang-Gung Memorial Hospital, Chang-Gung University College of Medicine, Taoyuan, Taiwan; 7 Department of Pathology, Tzu-Chi General Hospital, Taipei Branch, Tzu-Chi University School of Medicine, Hualien, Taiwan; 8 Department of Pathology, Tzu-Chi General Hospital, Dalin Branch, Chiayi, Taiwan; Johnson & Johnson Medical, China

## Abstract

**Background & Aims:**

The correlation between chronic hepatitis B virus (HBV) infection and hepatocellular carcinoma (HCC) has been well-established. But the roles of viral factor remain uncertain. Only HBV X gene and nonsense mutations of S gene (C-terminal truncation of HBV surface protein) have been demonstrated to have transforming activity. Whether they play a significant role in hepatocarcinogenesis is still uncertain.

**Methods:**

Twenty-five HBV-related HCC patients were positive for hepatitis B core antigen (HBcAg) in the cancerous parts of their HCC liver tissues by immunohistochemistry studies, and had available tissue for whole HBV genome sequence analysis. The results were compared with 25 gender and age-matched HBcAg negative HCCs. Plasmids encoding HBV S gene nonsense mutations identified from HBcAg (+) HCC tissue were constructed to investigate their cell proliferation, transformation activity and the oncogenic potentials by xenograft study and in vivo migration assay.

**Results:**

HBcAg (+) HCC patients were significantly associated with cirrhosis and small tumor size (≦2 cm) when compared with HBcAg (−) HCC patients. Southern blot analyses revealed freely replicative forms of HBV in the cancerous parts of HBcAg(+) HCC. Three nonsense mutations of S gene (sL95*, sW182*, and sL216*) were identified in the HBcAg(+) HCC tumor tissues. sW182* and sL216* were recurrently found in the 25 HBcAg (−) HCC tumor tissue, too. Functional studies of the above 3 non-sense mutations all demonstrated higher cell proliferation activities and transformation abilities than wild type S, especially sW182*. Tumorigenicity analysis by xenograft experiments and in vitro migration assay showed potent oncogenic activity of sW182* mutant.

**Conclusions:**

This study has demonstrated potent oncogenic activity of nonsense mutations of HBV S gene, suggesting they may play an important role in hepatocarcinogenesis.

## Introduction

Hepatocellular carcinoma (HCC) is one of the most common cancers and the leading cancer death in the world [1]. The high incidence of HCC in parts of Asia and sub-Saharan Africa are mainly related to the high prevalence of chronic hepatitis B virus (HBV) infection [2]. At the late stage of the disease, many of the HBV carriers will suffer from liver cirrhosis or HCC [3], [4]. The correlation between HBV infection and HCC has been well-established [3], [5], [6]. Since HBV accounts for 60% of liver cancers in the endemic area [1], the studies of HBV-related hepatocarcinogenesis were abundant, but the roles of viral factor remain controversial. HCC usually develops after a long time of chronic infection. Therefore, HBV is not an acutely transforming virus and multiple events should have been involved for the development of HCC. Previous studies have suggested that continuous and vigorous destruction and regeneration of hepatocytes might be the major mechanism of carcinogenesis, which could explain why most HCCs developed after cirrhosis [7], [8]. However, HCC develop without evidence of vigorous hepatocytes regeneration has also been reported, suggesting that viral factor alone might also be sufficient for hepatocarcinogenesis [9]. Thus, whether HBV could play a direct role in hepatocarcinogenesis without invoking chronic liver inflammation remained a subject of much debate.

HBV is a partially double-stranded 3.2 kb DNA virus. The genome of HBV contains four genes named PreS/S (surface), PreC/C (core), P (polymerase), and X. During the course of chronic HBV infection, the viral genome would develop mutations frequently. These mutations represent the attempt of the virus to escape from host immune-surveillance. In Taiwan, most chronic carriers of HBV were infected at birth through vertical transmission from their mothers, or infected during childhood [3], [4]. Consequently, a very high frequency of viral mutations was observed in adults, since these viruses have inhabited in the human host for a long time. Among the 4 genes of HBV, X gene has been reported to be a transcriptional transactivator and was able to positively regulate transcription of a wide variety of viral and cellular promoters [10], [11], [12]. It is the most well studied HBV gene regarding its relationship with hepatocarcinogenesis. Since the protein expression level of the X protein is very low in human liver or HCC tissues, it has been difficult to verify the role of X gene using human HCC tissues [13]. The P gene mutations were frequently associated with drug-resistance to the antiviral therapeutic reagents, such as lamivudine and other nucleoside analogues [14]. But it has not been reported to be associated with hepatocarcinogenesis. The PreC/C gene encodes a structural protein (capsid). No definite regulatory function has been reported. The PreS/S gene codes for the HBV surface antigen (HBsAg). The PreS/S gene is one long open reading frame but contains three in frame “start” (ATG) codons that divide the gene into three sections, pre-S1, pre-S2, and S. Because of the multiple start codons, polypeptides of three different sizes called large S, middle S, and small S (translated from pre-S1 + pre-S2 + S, pre-S2 + S, and S, respectively) are produced. Recently, PreS1/PreS2 deletion mutations have been suggested to be associated with hepatocarcinogenesis [15], [16], [17], but whether it is through immune evasion or direct oncogenic mechanisms is still uncertain. It has also been reported that nonsense mutation of HBV S gene, which resulted in C-terminal truncation of S proteins, could have transactivating function by Kekulé, et al. and Caselmann, et al. in 1990 [18], [19], [20]. But few studies were followed after these reports. In 2009, we reported 8 nonsense mutations of S gene identified in HBV patients who developed HCC after anti-viral treatment [21]. Additional functional studies demonstrated transformation activities in these mutants [21], which is similar to the reports by Kekulé, et al. and Caselmann, et al. [18], [19], [20].

From year 2000 to 2002, we have performed immunohistochemical (IHC) stains for hepatitis B surface antigen (HBsAg) and hepatitis B core antigen (HBcAg) on all surgically removed HBV-related HCC specimens in Chang-Gung Memorial Hospital and identified 28 HCCs which were positive for HBcAg in the cancerous parts, suggesting that HBV might still be viable in the HCC tissue. We believed that these tissues might preserve HBV mutants contributing to the early stage of hepatocarcinogenesis. In this study, whole HBV genome sequence analyses were performed on the 25 HBcAg(+) HCC patients with available tissue samples and compared with 25 gender and age-matched HBcAg(−) HCC patients to identify viral mutants replicating preferentially in the cancerous tissues. We identified 3 nonsense mutations of S gene in the tumor tissues of HBcAg(+) HCC. Additional functional studies of these mutations revealed transforming activities in all 3 mutations, especially the nonsense mutation at the codon for tryptophane182 (sW182*).

## Results

Among the 300 HBV-related HCC patients who received surgical operations for HCC from January 2000 to December 2002, there were 106 patients (35.3%) positive for HBsAg in the HCC tumor tissue by IHC stains, while only 28 patients (9.3%) were positive for HBcAg in the HCC tumor tissue (Figure S1A). Among the 28 HBcAg(+) HCC patients, only 25 patients had available fresh frozen tumor tissue and paired benign liver tissue in the tumor bank of CGMH for further studies. Fresh frozen tumor tissue and paired non-tumor liver tissues of 25 gender and age-matched HBcAg(−) HCC patients operated during the same period of time were also obtained for comparison.

### Identification of freely replicative HBV in HBcAg(+) HCCs

Among the 25 HBcAg(+) HCCs, only 19 tumors had sufficient amount of DNA samples (>100 ug) for Southern blot analyses. Freely replicative forms of HBV genomes were identified and isolated from the HBcAg(+) HCC tissues in all of the 19 HCC tumor tissue (Figure S1B).

### Identification of recurrent HBV mutations in HCC tumor tissues by whole genome HBV DNA sequence analyses

Whole genome HBV DNA sequencing was performed on all of the 25 HBcAg(+) HCC patients' paired tumor and non-tumor liver tissues. All of the four HBV genes (PreS/S, PreC/C, X and P) were successfully sequenced in the HBcAg(+) HCC tumor tissue. The complete HBV sequence data of the 25 HBcAg(+) HCC tumor and paired non-tumor tissue has been submitted to NCBI Nucleotide database (EU487256-EU487257, EU522066-EU522075, EU564820-EU564826, EU660224-EU660233, EU881995-EU882006, EU919161-EU919176) in year 2008, and had been released in 2009 and 2010. All mutations found in this study were heterologous, accompanied by co-existing wild type. Among the 4 genes, the three well-known mutation hot spots: nucleotide 1762 A → T, nucleotide 1764 G → A in core promoter region, and nucleotide 1896 G → A at PreC region were found in 7, 5 and 8 patients, respectively. Majority of these mutations were found in both tumor and paired non-tumor tissue. Other mutations in X gene, PreC/C gene, P gene, PreS1 and preS2 genes were all sporadic. Most were non-recurrent and found in both tumor and paired non-tumor tissue. Three nonsense mutations of S gene were identified (sL95*, sW182*, and sL216*) in the HCC tumor tissue of 4 patients. Among them, sW182* was found in two tumors and also identified in one of the paired non-tumor liver tissue.

Whole HBV DNA sequencing of 25 gender and age-matched HBcAg (−) HCCs were also performed. But the sequencing was not successful in many patients, especially the HCC tumor tissues. The sequencing of the PreC/C gene failed in all tumor tissues and was successful in only half of the paired non-tumor liver tissue. Sequencing for the X gene, P gene, and PreS/S gene was successful in most patients, but the sequences were very complex and difficult to read due to presence of multiple mutants, especially for PreS/S gene. For the nonsense mutation of S gene, a total of 8 mutations were found in the HCC tumor tissue, and 2 of them were found in the paired non-tumor liver tissue. Again, sW182* was the most frequently found mutations, being found in 4 tumors. The summary of the nonsense mutations of S gene identified in the 50 tumor tissues is shown in Table S1.

### Clinicopathological characteristics of HBcAg(+) HCC patients compared with HBcAg(−) HCC patients

To understand the clinical significance of positive HBcAg in HCC tissue, we have compared the clinicopathological characteristics of the 25 HBcAg(+) HCC patients with 25 gender and age-matched HBcAg(−) HCC patients operated in the same time period. The result is shown in Table S2. HBcAg(+) HCC patients were significantly associated with cirrhosis (p<0.001), and small tumor size (≦2 cm) (p = 0.039). The median disease free survival (DFS) and overall survival (OS) for HBcAg(+) HCC patients (21.2 months and 72.2 months, respectively) were all longer than HBcAg(−) patients (9.8 months and 44.0 months, respectively), but both were statistically non-significant (P = 0.686 and 0.793, respectively). The Kaplan-Meier curves of DFS and OS are shown in Figure S2. Multivariate analysis for the survivals was performed using Cox's regression model. The variables for the multivariate analyses included age, gender, cirrhosis status, tumor size, tumor grade and tumor stage.

### Transformation and tumorigenesis assays of the 3 non-sense mutations of HBV S gene

#### 1) Cell proliferation assays

The WST-1 reagent was used for cell proliferation assay. The proliferation assay of the 4 stable clones: wild type (Wt), sL95*, sW182* and sL216*, indicated that all three nonsense mutations of S gene could enhance the cell growth compared with Wt and Mock (Figure S3A).

#### 2) Cell anchorage independence abilities

Colonies of 4 stable clones: the Mock, Wt, sL95*, sL216*, and sW182*, in soft agar were visualized by 0.1% *p*-iodonitro tetrazolium violet (*INT*) staining. The average colony numbers counted by the Soft ImageJ (NIH) were shown (Figure S3B). Wt and three mutants all had significantly higher colony counts than Mock. Among the three mutants, sW182* had the highest colony counts (Figure S3C).

#### 3) The xenograft study

Xenograft studies were performed using 4 weeks old male athymic BALB/c nude mice with subcutaneous injection of 1×10^6^ pIRES-preS/S-wt and pIRES-preS/S-mut stable clone cells, which included Mock, Wt-1, Wt-2, and four nonsense mutations of S gene:L95, L216, W182–1, and W182–2 at the same time. Tumor growth was monitored and measured weekly until week 13. Wt and all three mutants had tumor growth in some or all mice. Only the W182–1 (100%) and W182–2 clones (100%) had significant higher tumor growth rate compared with Mock, and the tumor sizes of sW182* clones were also the largest compared with Wt and the other two mutants (Figure S4). The RNA expression of HBS gene was all positive in the stable clones (except Mock) and the tumors of xenograft study (Figure S5A and S5B). For xenograft study, the protein (HBV surface antigen) expression level was evaluated by immunohistochemical stains on all tumors in the xenograft study (Figure S5C). The HBV surface antigen expression was lowest in the tumors derived from sL216*. Tumors derived from this clone had smallest tumor size and only 50% of the nude mice had tumor growth (2/4). The above results suggested that HBV surface antigen expression level might affect the tumor growth in this xenograft study.

#### 4) *In vivo* migration assay by a zebrafish xenotransplantation model

Three stable clones: vector-only, Wt and W182 were used for this *In vivo* migration assay. The cells of stable clones had been labeled with DiI cell label solution for observation of their migration. On the first day post-injection (1 dpi), 13%of the W182 cells already migrated to the trunk. On the 3rd day post-injection (3 dpi), 46% of W182 cells had migrated to the trunk and tail (Figure S6A and S6B). In contrast, almost all of the injected cells of the Wt and control (vector-only) still stayed in the yolk at 3 dpi (Figure S6C and S6D). W182 exhibited significantly higher migration ability compared to Wt or vector-only (Figure S6E).

### Western blot assay for seven signaling molecules

Phosphorylation profile of signaling molecules in 3 stable clones: Wt, vector only and sW182* mutant, were evaluated for 7 proteins: (a) ERK. (b) JNK. (c) JAK1. (d) JAK2. (e) Stat1. (f) Stat3. (g) Stat5. sW182* mutants showed higher phosphorylation level than Mock and Wt in most of the proteins except ERK and Stat1 (Figure S7 A to G).

### Assessment of endoplasmic reticulum (ER) stress

ER stress assessment revealed no evidence of ER stress in the 3 HBV mutants, and Wt stable clones (Figure 7H).

## Discussion

We have identified a small percentage of HCCs with positive staining of HBcAg in the cancerous tissue by IHC stain. Since this IHC staining result usually suggested active replication of HBV, we have performed southern blot analyses for HBV and confirmed the presence of freely replicating HBV in cancerous tissues, which was rarely identified before. The successful sequencing of the PreC/C gene in all 24 HBcAg(+) HCC tumor tissues was another strong supporting evidence to suggest that they were not derived from integrated HBV DNA. It is well known that HBV DNA frequently becomes fragmented after integration into the genome of hepatocytes [22]. In addition, the insertion points of HBV usually are located near or on the PreC/C gene region [23]. Therefore, in the integrated phase of chronic HBV hepatitis, sequencing of the PreC/C gene in liver tissue would commonly fail. This could explain why sequencing of PreC/C gene failed in all tumor tissues of the HBcAg(−) HCCs and was successful in only half of the paired non-tumor liver tissue. The HBcAg(+) HCCs in this study were significantly associated with smaller tumor size when compared with the gender and age-matched HBcAg(−) HCCs, suggesting they were in the earlier tumor stage. Thus, among these freely replicating HBV in the cancerous tissues, HBV mutants contributing to the early stage of hepatocarcinogenesis might still present. Hepatocarcinogenesis of HBV-related HCC is believed to be a “hit-and-run” event as most of the cancerous HCC tissues when surgically removed, contain rare, if any, freely replicative viruses [22]. Therefore, if certain viral mutations were assumed to be responsible for initiation of the oncogenic process, one could not identify them. Since the mutant viruses should have been lost in the subsequent steps of hepatocarcinogenesis. We therefore took advantage of this opportunity to identify viral mutants replicating preferentially in the cancerous tissues. These mutants were either selected by the cancerous microenvironment or alternatively, they maintained the continuous oncogenic process of the liver cancer. Among the 25 HBcAg(+) HCCs that we were able to perform whole HBV genome sequencing, majority of the mutations were either random or found in both tumor and paired non-tumor liver tissue. We did not identify any recurrent mutations of X gene that were associated with cancerous part. On the other hand, three nonsense mutations of S gene were found in the cancerous part (sL95*, sW182* and sL216*). Among them, sW182* was found in 2 tumors with one identified in the noncancerous parts, too. When we performed whole HBV genome sequencing on the 25 gender and age-matched HBcAg(−) HCCs, additional nonsense mutations of S gene were found in the cancerous part, and sW182* was recurrently found in the tumor tissue (6 of the 50 HCCs). We therefore performed various transformation activity studies on these 3 nonsense mutations of S gene. The study results demonstrated the potent oncogenic activity of the sW182* mutant, especially the xenograft study and in vivo migration assay (Figure S4 and S5). The other two S truncation mutants, sL95* and sL216*, also found to had transformation activities, though weaker than sW182*.

Since there is no available benign or normal liver cell line for transformation study, the stable clones used in this study were NIH3T3 cell line (mouse fibroblast), which is considered as a standard cell line for testing the transformation activities. Thus, transgenic mice study will be ideal for further confirmation, which is underway now. Recently, a study from Korea by Lee, et al. also reported that sW182* mutation was associated with progression of liver disease in genotype C HBV patients [24]. They found a significantly higher incidence of sW182* mutation in HBV patients with cirrhosis or HCC (37.8%), when compared with chronic hepatitis or carriers (17.2%). They also have performed functional study of sW182* mutation, including cell growth, cell cycle regulation, and cell transforming ability by using NIH3T3 cell line. The results demonstrated that the cell growth, cell cycle progression and cell transforming abilities were all significantly increased in the sW182* expressing stable clone, when compared with mock and wild type clones, which was quite similar to our results. In addition, they also found similar results in the HuH-7 and HEK 293 stable clone cells with sW182* mutation. The main difference between our study and Lee, et al. was the incidence of sW812* mutation. In our study, the incidence of sW182* was quite low (6/50, 12%) in HCC cancerous tissue samples, while the incidence was rather high in the study by Lee, et al. (35/113, 30.9%) in the blood samples of genotype C HBV patients with HCC. All mutations in our study were detected by direct sequencing and from liver tissue, while in the study by Lee, et al. the mutations were detected by real time PCR on blood samples. The high incidence by Lee, et al. might be due to the detection method (real time PCR) or ethnic background. For blood sample, the HBV DNA level might also affect the detection rate. We also have performed HBV S gene sequencing in the serum sample of 64 genotype C HBV-related HCC patients, and the incidence of sW182* (4/64, 6.25%) was lower than from HCC cancerous tissue (preliminary unpublished data).

Because the HBS truncation mutation was developed from the wild type and thus the mutants usually coexisted with the wild type in the viral population. In some patients, the S truncation mutants could become the dominant species due to unrecognized selection pressure or survival benefit in HCCs. However, in such cases, a very small percentage of coexisting wild type virus can still be present. As such, it is difficult to identify the phenotypic characteristics of HCCs specific to the sW182* mutation. Clinical analysis by comparing the tumor characteristics in HCC patients with and without sW182* mutation showed no difference (data not shown), since these tumors should all have gone though highly complex processes in the cancer development by the time of resections.

In this study, two of the 6 HCC patients harbored the sW182* mutation.in their tumor tissues also had the same mutation in the non tumor parts, suggesting this mutation occurred before the HCC development. Together with the 3 nonsense mutations in our previous report (sL21*, sW156*, sW172*) [21], we already found 6 different HBV S nonsense mutations with transformation activities. It is possible that most of the S gene nonsense mutations had transformation activity. Thus, it was highly likely that such mutants played a role in the initiation stage of liver cancer cell transformation. However, judging from the persistence of this mutant in the cancerous tissue, it was possible that the mutant also contributed to maintenance of HCC tumor growth. Furthermore, we discovered that 3 tumor tissues with sW182* had multiple S nonsense mutations (Table S1), which might result in an additive effect to enhance their transformation ability.

Among the 11 HBV S gene truncation mutants we have identified (sL15*, sL21*, sS61*, sC69*, sL95*, sW156*, sW163*, sW172*, sW182*, sW196*, and sL216*) in HBV-related HCCs, all except one (sL95*) were belonged to genotype C of HBV, which is also quite interesting, since genotype C has been found to be one risk factor for development of HBV-related HCC in Taiwan [25].

In summary, this study has demonstrated potent oncogenic activity of nonsense mutations of HBV S gene, suggesting they may play an important role in hepatocarcinogenesis. Identification of HBV S gene nonsense mutations in a larger population of chronic HBV patients is ongoing to test our hypothesis.

## Materials and Methods

### Patients and tissues

From year 2000 to 2002, routine IHC stains for HBsAg and HBcAg on all surgically removed HBV-related HCC liver tissue sections in Chang-Gung Memorial Hospital (CGMH). The results of IHC stains were useful for future antiviral treatment. Fresh frozen HCC tumor tissues and paired benign liver tissue with signed informed consent of 50 HBV-related HCC patients with the above HBsAg and HBcAg information were obtained from the tumor bank of CGMH for further studies on HBV mutations and clinical correlations. The histopathology and tumor percentage of all fresh frozen liver tumor tissues were examined by frozen sections stained with hematoxylin and eosin first. In all cases studied, the tumor tissue all had tumor percentage higher than 90%, and all paired non-neoplastic liver tissue had no tumor contamination. The clinical data were obtained from medical records. This study protocol was approved by the Institutes of Reviewing Board of Chang-Gung memorial Hospital and National Health Research Institutes.

### Immunohistochemical (IHC) stains for HBsAg and HBcAg

IHC stain for HBsAg and HBcAg were performed on unstained 4-μm-thick paraffin-embedded formalin-fixed tissue sections. After deparaffinization by Xyline, the slides were incubated with primary antibody for HBsAg (Virostat, ME, Portland) and HBcAg (Dako, CA, USA) with a dilution of 1∶200.The detection was performed by the Ventana Discovery XT staining system with ultraView Universal DAB detection kit (Ventana Medical Systems, Inc, Tucson, AZ) according to the manufacturer's protocol.

### Identification of HBV mutations

DNA extraction was performed using Hirt extraction procedure followed by Quiagen DNA extraction (QIAGEN, Hilden, Germany). The method of Hirt extraction and southern blot analysis has been described previously [26].

### Nucleotide sequence of full-length HBV genomes

For sequencing analysis, we used 10 pairs of primer to cover full-length of the HBV genome (Table S3). The PCR amplicons were subjected to direct sequencing. Forward and reverse sequencing reactions were done with the same primers for PCR amplification and ABI BigDye Terminator kit v3.1 on an ABI3730 genetic analyzer (Applied Biosystems, Foster City, CA) according to manufacturer's instructions. Sequence variations were determined by using Seqscape software (Applied Biosystems, Foster City, CA, USA) with the Hepatitis B virus reference sequence (AY167089 for genotype B and AY167095 for genotype C, National Center for Biotechnology Information).

### Transforming activity of S gene C-terminal truncation mutants

#### 1) Plasmid construction and site directed mutagenesis

The wild type (wt) preS/S region of HBV was amplified from pCMV-HBV plasmid, which contained longer-than-one copy of HBV genome (GenBank accession number X02763), using the PreSF and SR primers. The PCR product was inserted into pIRESbleo plasmid (BD Biosciences Clontech, NJ, USA) to generate pIRES-preS/S-wt. To construct plasmids encoding truncated preS/S proteins containing 3 nonsense mutations, sL95*, sW182* and sL216*, respectively, site directed mutagenesis experiments were preformed according to a PCR-based method [22]. All plasmids were sequence verified using an automatic DNA sequencer (Applied Biosystems, CA, USA).

#### 2) Cell culture and plasmid transfection for establishment of the stable clones of three S truncation mutants in NIH/3T3 cells

NIH3T3 cells were maintained in Dulbecco's modified Eagle's medium supplemented 10% calf serum, 2 mM glutamine, 100 unit/ml penicillin and 100ug/ml streptomycin. Cells were maintained at 37°C in a 5% CO_2_ incubator. Transfection was performed using Lipofectamine 2000 reagent (Invitrogen, CA, USA). After Zeocin selection, some stable cell clones were isolated for “vector”, “wild type” and “mutant”, respectively. These cells were maintained with occasional treatments with 0.1mg/ml Zeocin (Invitrogen, Carlsbad, CA, USA).

#### 3) Cell proliferation assays

The WST-1 reagent was used for cell proliferation assay. It was diluted (1∶10) in culture media. One thousand cells were seeded in each well of a 96-well culture plate, and incubated for 12 hours. After cell attachment, the culture media were changed to the media with no sera, then incubated for 24 hours for synchronization. After Day 0 data were collected, culture media were changed back to complete media and renewed every two days. After 4 hours incubation, the optical densities of wave length 450 nm were read. Each detecting point was analyzed in 5 independent experiments. The growth curves were calculated as the ratios of absorbance readings in Day N to that in Day 0 of the tested stable clones.

#### 4) Cell anchorage independence abilities

The cell anchorage independence assays were performed in 6-well plates. The bottom layer was 1.5 ml 0.7% agarose (SeaPlaque) with complete culture media. The upper layer was 2 ml 0.5% agarose mixed with complete culture media and 5000 cells. The top layer was 1 ml complete culture medium and refreshed every 2 days. Colonies were visualized by 0.1% *p*-iodonitro tetrazolium violet (*INT*) staining. Each stable clone experiment was performed in triplicate and 3 randomly selected fields for each well was subjected for colony number calculation in the same objective magnification (20X). The colony number was counted by the soft ImageJ (NIH).

#### 5) The xenograft assays for tumorigenicity

Male BALB/c mice were obtained from the National Animal Experimental Center (Taipei, Taiwan). The mice were maintained in specific pathogen-free conditions. Four-week-old athymic mice (4–5 per group) were injected subcutaneously with 1×10^6^ pIRES-preS/S-wt or pIRES-preS/S-mut cells. Tumor growth was monitored and measured weekly until week 13. The mice were then sacrificed. If there was tumor growth, the tumor and liver were excised, formalin-fixed and paraffin-embedded for histopathology examination. Portions of the tumor and liver tissue were frozen for DNA, RNA and protein extraction. If there is no tumor growth grossly, the skin and its subcutaneous tissue of the injection site were excised for histopathology confirmation. The animal use protocol had been approved by the Institutional Animal Care and Use Committee of National Health Research Institutes.

#### 6) *In vivo* migration assay by a zebrafish xenotransplantation model

*In vivo* migration assay was performed to evaluate the tumor cell dissemination and migration behavior using a zebrafish xenotranplantation model according the previous reports [27], [28]. Briefly, the cells of stable clones in suspension were labeled with DiI cell label solution (Vybrant^TM^Cell-Labeling Solutions, Invitrogen V-22885) and resuspend in PBS (PH 7.4) with a final concentration of 9×10^4^ cells/μl. Before microinjection, the 2 days old embryos from *Tg(fil1:GFP)* zebrafish was anesthetized by placing them briefly in a fish tank with fish water containing 0.048% Tricaine (MS-222, Sigma-Aldrich, USA) for a few minutes. About 400 cells were injected into the yolk of 2 dpf embryo. Microinjection was done using Nanoject II (Drummond Scientific Company, USA) with capillaries of 3.5??Drummond #3-000-203-G/X (Drummond Scientific Company) pulled by a FLAMING/BROWN MICROPIPETTE PULLER MODEL P-97 (Sutter Instrument Co.) in fixed parameter (heat 607, pull 180, velocity 150, delay time 100 and pressure 500). Injection was carried out under microscope Leica EZ4 16x (LEICA). After 2 hours post injection, the embryos with labeled cells were removed, and kept in 28°C incubators for observation of migration behavior under microscope for 3 days continuously. The transparency of zebrafish embryos and transparent adult fish allow us to monitor tumor progression and migration behaviour under real-time confocal microscope. The assay was repeated for 3 times.

### Assessment of extracellular signal-regulated kinase (ERK), c-Jun N-terminal kinases (JNK), Janus kinase (JAK), and signal transducers and activators of transcription (STAT) signal pathways by Western blot analyses

The cell extracts from stable clone were examined by western blot. The phosphorylation of the following proteins: extracellular signal-regulated kinase (ERK), c-Jun N-terminal kinases (JNK), Janus kinase 1 (JAK1), JAK2, signal transducers and activators of transcription 1 (Stat1), Stat3 and Stat5, were performed. The antiphospho-ERK, -JNK, -JAK1, -JAK2, -Stat1, -Stat5 and anti- JNK, -JAK1, -JAK2 antibodies were purchased from Cell Signaling Technology (Beverly, MA, USA). Antibodies for Stat1, Stat3, Stat5 and phospho-Stat3 were purchased from Santa Cruz Biotechnology (Santa Cruz, CA, USA). Anti-ERK antibody was purchased from Millipore (Billerica, MA, USA). Antibody for β-actin was purchased from Sigma-Aldrich (Saint Louis, MO, USA).

The cells of stable clone were first trypsinized, then lysed in lysis buffer (50 mmol/L Tris (pH 7.4), 150 mmol/L NaCl, 1% Triton X-100, 0.5% sodium deoxycholate, 0.1% (w/v) SDS, 1 mmol/L dithiothreitol, 5 μg/mL leupeptine, 3 g/mL aprotinin, 0.5 mmol/L phenylmethylsulfonylfluoride, and 1mmol/L Na_3_VO_4_) with the MagNA Lyser Green Beads kit (Roche Applied Science, Mannheim, Germany), and centrifuged at 14,000 g to remove the tissue debris. Protein concentrations were determined by Bradford protein assay and fifty micrograms of extracted protein were loaded per lane of 8% SDS–PAGE for electrophoresis, and then transferred to polyvinylidene difluoride (PVDF) membrane (Millipore, Bedford, MA, USA). After blocking in TBST buffer (20 mM Tris (pH 7.5), 135 mM NaCl, and 0.1% Tween 20) with 5% milk, the membrane was incubated with a primary antibody (dilution of antibodies were performed according to the manufacturer's instructions), washed, and blotted with secondary antibody conjugated with horseradish peroxidase (1∶2000 dilution). β-actin was used as the internal control. The membrane was then developed in ECL reagents: Western Lighting Plus (PerkinElmer, Waltham, USA), SuperSignal West Pico Luminol/Enhancer (Pierce, Rockford, IL, USA) or SuperSignal West Femto Luminol/Enhancer (Pierce, Rockford, IL, USA)] and exposed to X-ray films.

### Assessment of endoplasmic reticulum (ER) stress

RNA was extracted from stable transformants and reverse transcription was performed using the oligo-dT primer. Spliced and unspliced forms of mouse X box binding protein-1 (XBP-1) mRNA were detected by RT-PCR. The primers used were 5′-GTTAAGAACACGCTTGGGAATGG-3′ (sense) and 5′-TGTCAGAGTCCATGGGAAGATGTT-3′ (antisense). As a positive control, cells were treated with brefeldin A (1 mg/ml) for 24 h before assay.

### Statistical analysis

To examine the differences in the major clinicopathological features associated with HBcAg(+) HCC or sW182* mutations, the frequencies and proportions are compared by conventional chi-square association test or Fisher's exact test (when there is at least a cell frequency less than 5). A two-sided *p* value less than 0.05 was considered statistically significant. The differences in disease free survivals and overall survivals were examined by log-rank tests. Multivariate analysis for the survivals was performed using Cox's regression model.

## Supporting Information

Figure S1 **Hepatitis B viruses (HBV) identified in the hepatocellular carcinoma (HCC) tumor tissue.** A. Diffusely positive staining for hepatitis B surface antigen (HBsAg) and hepatitis B core antigen (HBcAg) in one HBcAg(+) HCC (Immunohistochemical stain, 400X). B. Southern blot analyses of 19 HBcAg(+) HCC tumor tissue revealed abundant single-strand (SS) form and relaxed- circular (RC) forms of HBV, which was consistent with presence of freely replicative HBV.
(TIF)

Figure S2 **The Kaplan-Meier curves of A. disease free-survival and B. overall survival, of the 25 HBcAg- positive hepatocellular carcinomas (HCC) versus 25 HBcAg- negative HCCs.**
(TIF)

Figure S3 **Functional studies of three HBs mutants compared with wild type (WT).** A. The proliferation assay of the 4 stable clones: wt, sL95*, sW182* and sL216*, indicated that all three S truncation mutants could enhance the cell growth compared with wt and Mock. B. Cell anchorage independence ability study revealed sW182* had the highest colony counts. C. Bar figure exhibited that Wt and the three mutants all had significantly higher colony counts than Mock. The star sign (*) represents statistically significant when compared with Mock.
(TIF)

Figure S4 **The nude mice xenograft study by subcutaneous injection of the cells from the stable clones, which included 3 HBV S truncation mutants (L95, W182–1, W182–2 and L216), wild type (Wt-1, Wt-2), and Mock.** A. The W182 mutant showed the highest incidence (100%) and largest tumor sizes. B. Bar figure showed that only W182 mutant had statistically significant higher tumor growth rate compared with Mock. The star sign (*) represents statistically significant when compared with Mock.
(TIF)

Figure S5 **The m-RNA expression and protein expression of HBV S gene in the HBS stable clones and xenograft.** A. m-RNA expression of HBV S gene was all positive in the 5 stable clones of HBV S gene, including wild type (Wt), and truncation mutants: L95, L216, W182–1 and W182–2. B. The tumors of xenograft study from Wt and two W182 stable clones all had m-RNA expression of HBV S gene. C. Tumors in xenograft study from Wt and the All HBS truncation mutants all had HBS protein expression by Immunohistochemical stains (Immunohistochemical stain for HBsAg, 400X).
(TIF)

Figure S6 **In vivo migration assay of three stable clones: W182 mutant, wild type (Wt) and control (vector-only) by a zebrafish xenotransplantation model.** At the third day post-injection (3 dpi): A. W182 cells already had abundant cells migrated from yolk (arrow) to the trunk (open arrow), and B. tail (arrow head). C. The Wt cells all still stayed in the yolk (arrow). D. The vector-only cells also still stayed in the yolk (arrow). E. Bar figure exhibited significantly higher migration ability of W182 mutant compared to wild-type and control since the first day post injection.
(TIF)

Figure S7 **Phosphorylation profile of signaling molecules in 3 stable clones: Wt, vector only, and W182 were evaluated for 7 proteins: (A) ERK. (B) JNK. (C) JAK1. (D) JAK2. (E) Stat1. (F) Stat3. (G) Stat5.** W182 mutants showed higher phosphorylation level than MOCK and Wt in most of the proteins except ERK and Stat 1. (H) Assessment for endoplasmic reticulum (ER) stress showed no splicing of mouse X box binding protein-1 (XBP-1) for all 3 HBV S truncation mutants (L95, L216, W182), and the Wt, which meant negative for ER stress.
(TIF)

Table S1 **Nonsense mutations of Hepatitis B virus S gene in 50 HBV-related HCC patients.**
(DOCX)

Table S2 **Clinical Characteristics of the 25 HBcAg(+) HCC and 25 matched HBcAg(+) HCC patients.**
(DOCX)

Table S3 **Primer sets for detection of the full-length Hepatitis B Virus DNA genome.**
(DOCX)
