# Supplementary figures and images for: Identification of Transforming Hepatitis B Virus S Gene Nonsense Mutations Derived from Freely Replicative Viruses in Hepatocellular Carcinoma
Source: PLoS One. 2014 Feb 24;9(2):e89753. doi: 10.1371/journal.pone.0089753 (PMC3933656; doi:10.1371/journal.pone.0089753)

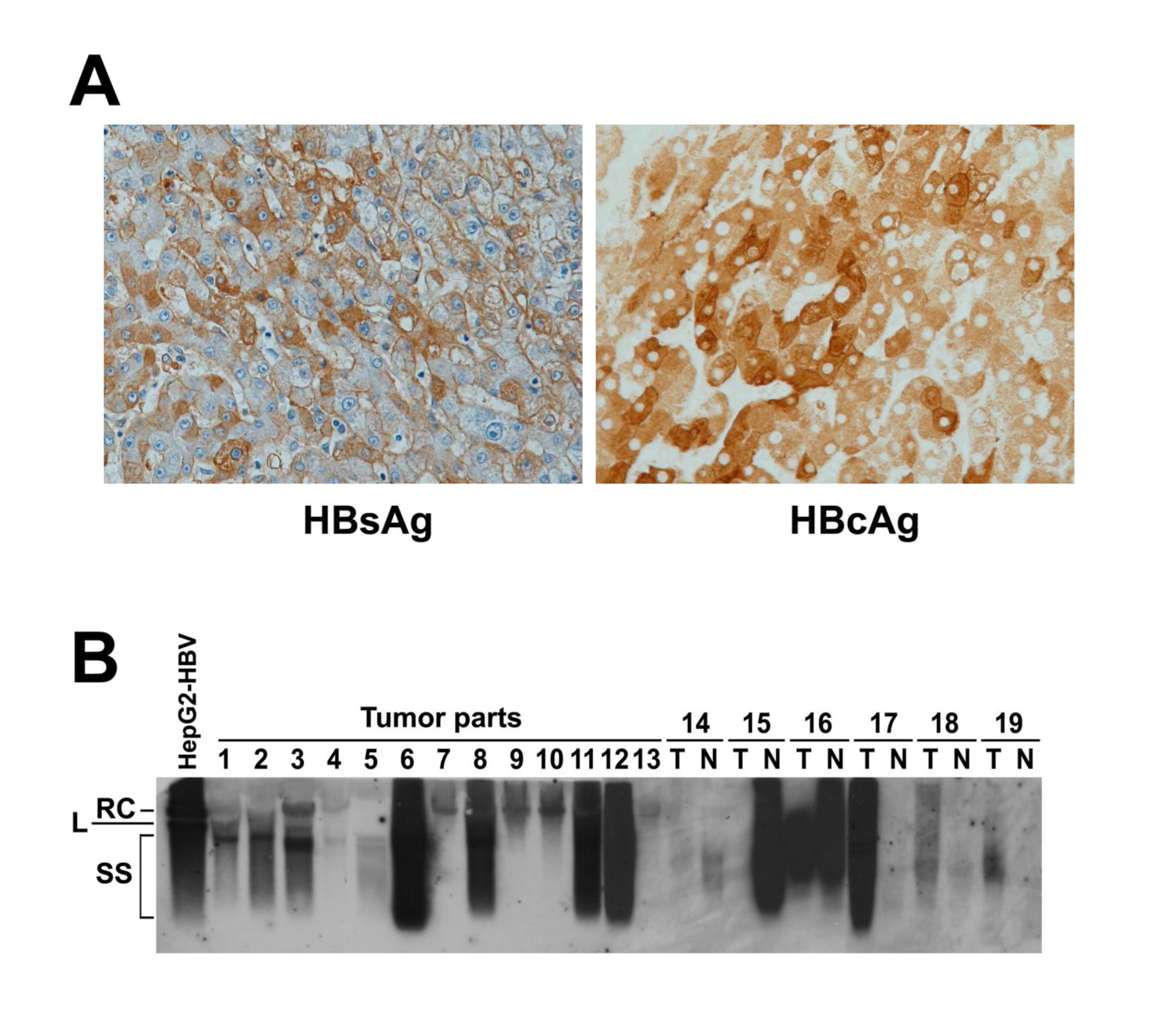

Supplement: Figure S1 — Hepatitis B viruses (HBV) identified in the hepatocellular carcinoma (HCC) tumor tissue. A. Diffusely positive staining for hepatitis B surface antigen (HBsAg) and hepatitis B core antigen (HBcAg) in one HBcAg(+) HCC (Immunohistochemical stain, 400X). B. Southern blot analyses of 19 HBcAg(+) HCC tumor tissue revealed abundant single-strand (SS) form and relaxed- circular (RC) forms of HBV, which was consistent with presence of freely replicative HBV. (TIF) [file pone.0089753.s001.tif]

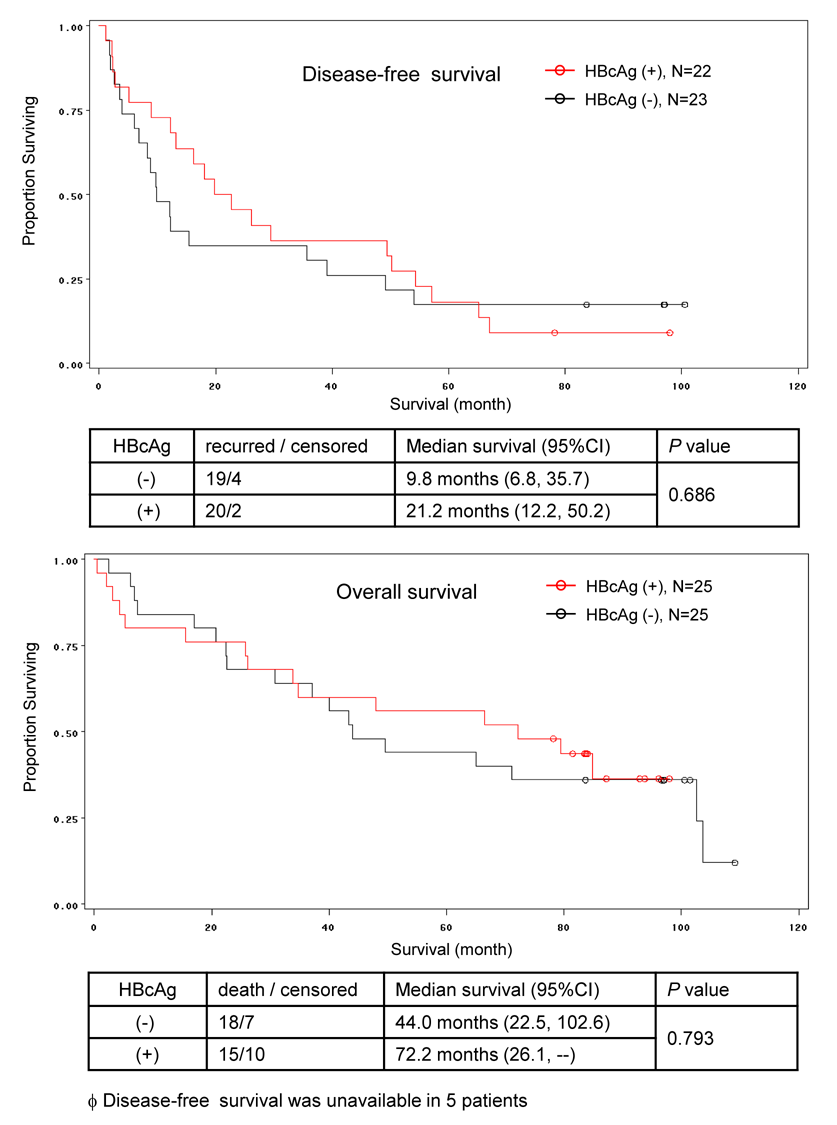

Supplement: Figure S2 — The Kaplan-Meier curves of A. disease free-survival and B. overall survival, of the 25 HBcAg- positive hepatocellular carcinomas (HCC) versus 25 HBcAg- negative HCCs. (TIF) [file pone.0089753.s002.tif]

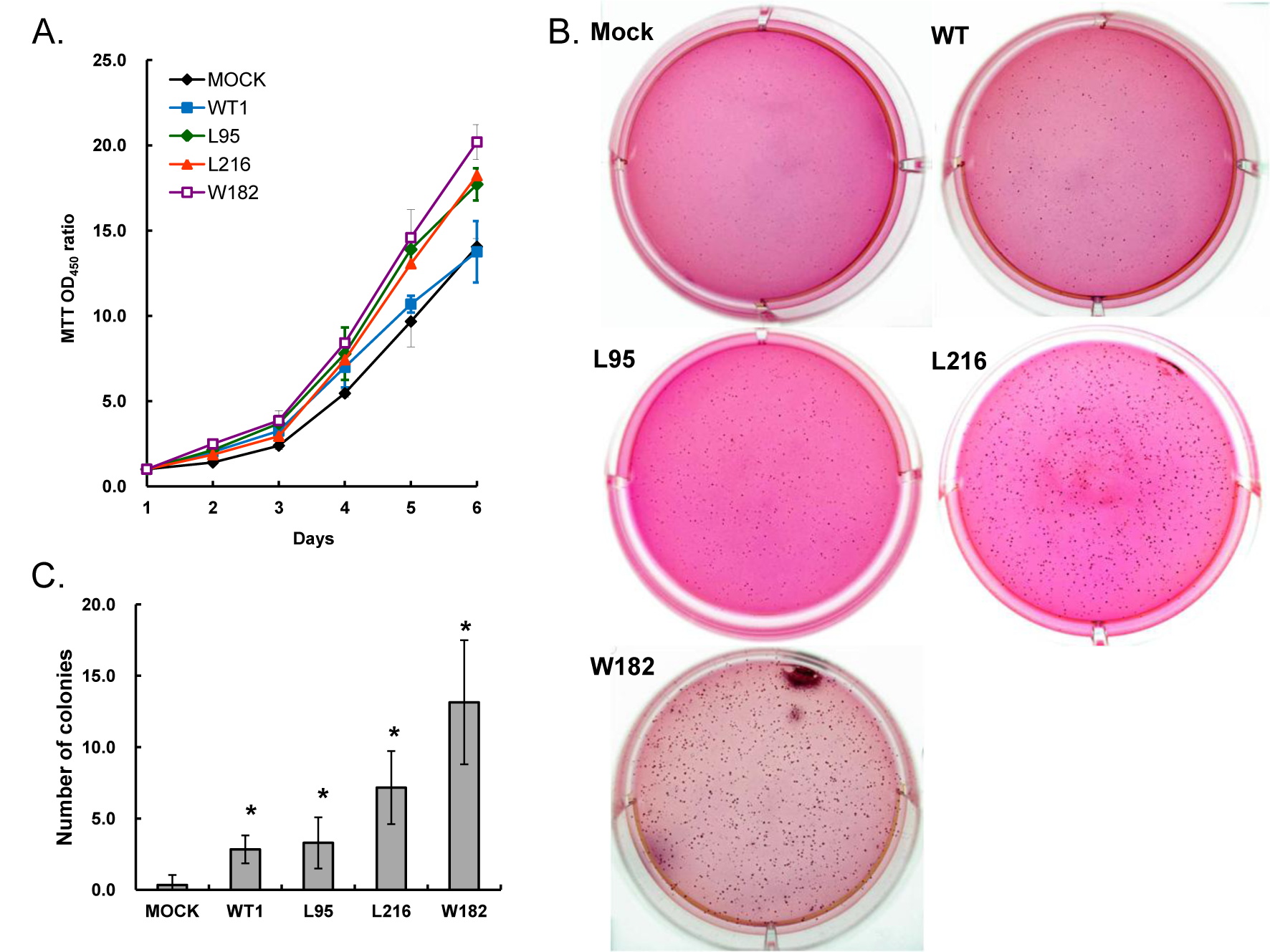

Supplement: Figure S3 — Functional studies of three HBs mutants compared with wild type (WT). A. The proliferation assay of the 4 stable clones: wt, sL95*, sW182* and sL216*, indicated that all three S truncation mutants could enhance the cell growth compared with wt and Mock. B. Cell anchorage independence ability study revealed sW182* had the highest colony counts. C. Bar figure exhibited that Wt and the three mutants all had significantly higher colony counts than Mock. The star sign (*) represents statistically significant when compared with Mock. (TIF) [file pone.0089753.s003.tif]

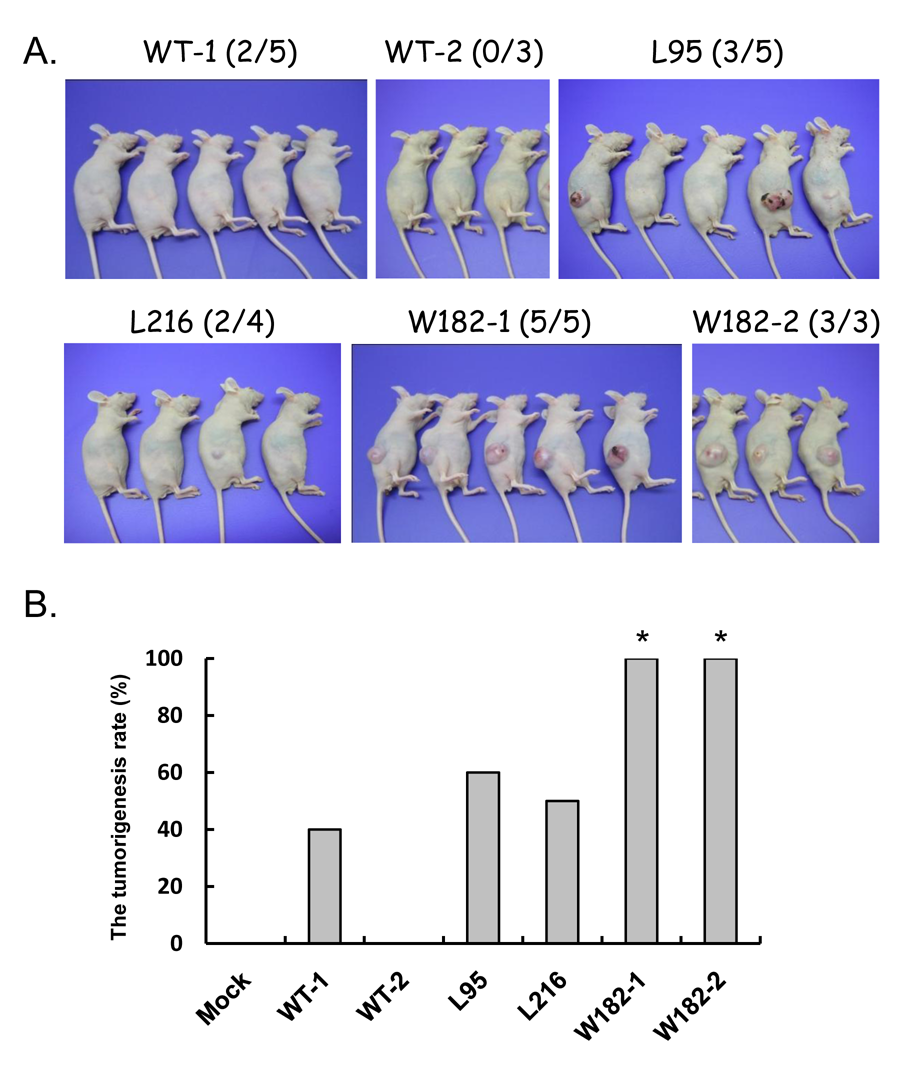

Supplement: Figure S4 — The nude mice xenograft study by subcutaneous injection of the cells from the stable clones, which included 3 HBV S truncation mutants (L95, W182–1, W182–2 and L216), wild type (Wt-1, Wt-2), and Mock. A. The W182 mutant showed the highest incidence (100%) and largest tumor sizes. B. Bar figure showed that only W182 mutant had statistically significant higher tumor growth rate compared with Mock. The star sign (*) represents statistically significant when compared with Mock. (TIF) [file pone.0089753.s004.tif]

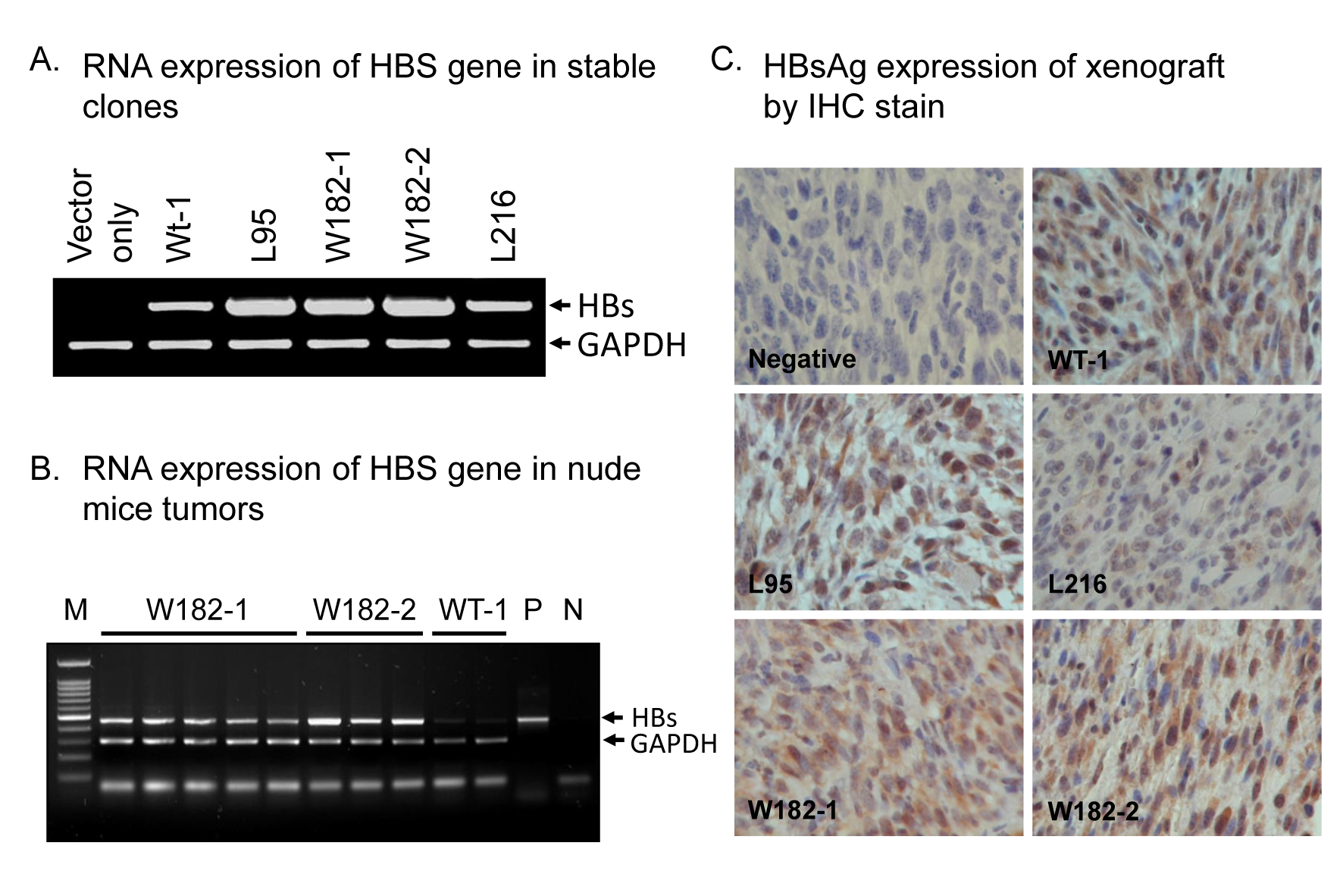

Supplement: Figure S5 — The m-RNA expression and protein expression of HBV S gene in the HBS stable clones and xenograft. A. m-RNA expression of HBV S gene was all positive in the 5 stable clones of HBV S gene, including wild type (Wt), and truncation mutants: L95, L216, W182–1 and W182–2. B. The tumors of xenograft study from Wt and two W182 stable clones all had m-RNA expression of HBV S gene. C. Tumors in xenograft study from Wt and the All HBS truncation mutants all had HBS protein expression by Immunohistochemical stains (Immunohistochemical stain for HBsAg, 400X). (TIF) [file pone.0089753.s005.tif]

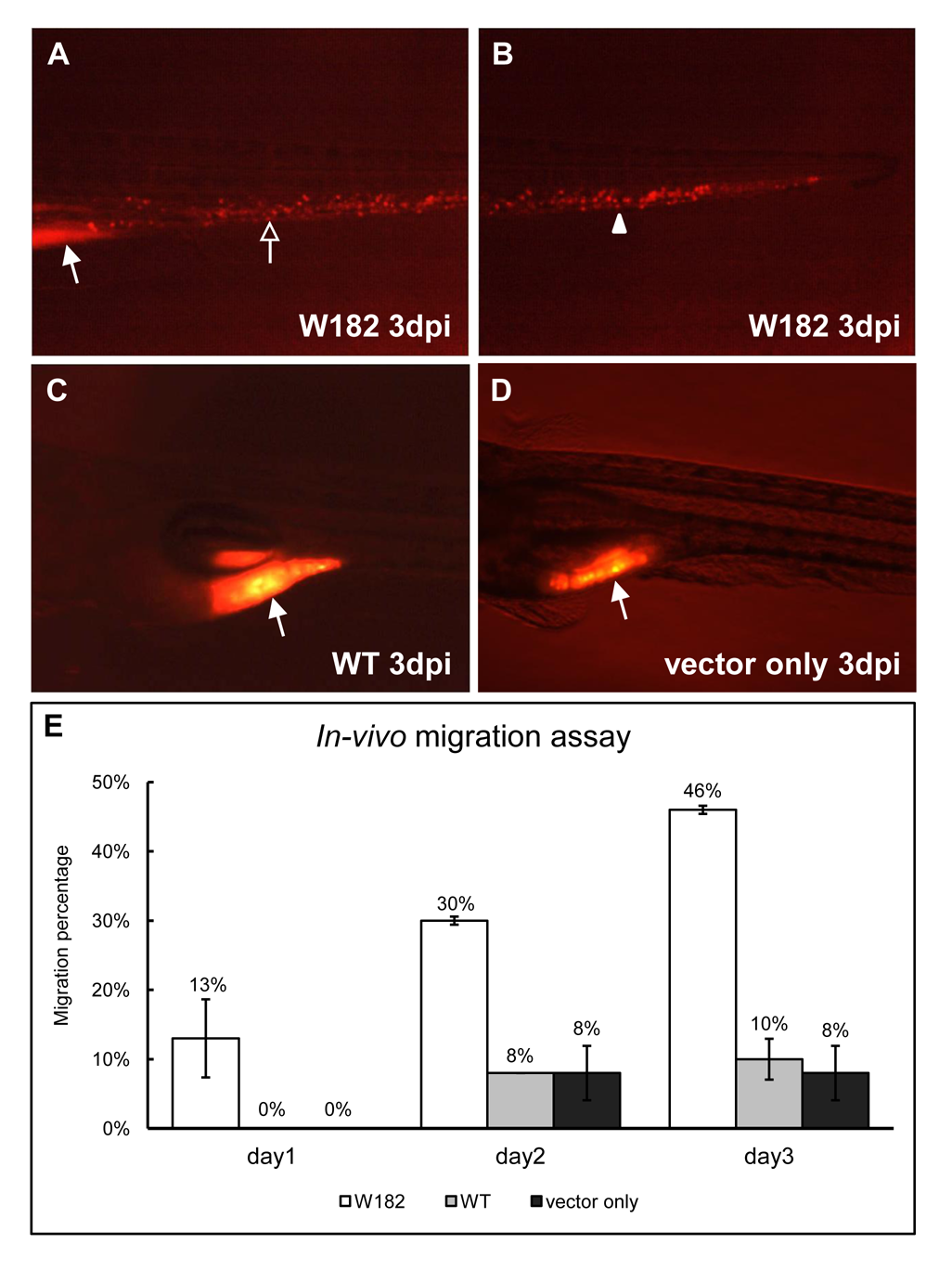

Supplement: Figure S6 — In vivo migration assay of three stable clones: W182 mutant, wild type (Wt) and control (vector-only) by a zebrafish xenotransplantation model. At the third day post-injection (3 dpi): A. W182 cells already had abundant cells migrated from yolk (arrow) to the trunk (open arrow), and B. tail (arrow head). C. The Wt cells all still stayed in the yolk (arrow). D. The vector-only cells also still stayed in the yolk (arrow). E. Bar figure exhibited significantly higher migration ability of W182 mutant compared to wild-type and control since the first day post injection. (TIF) [file pone.0089753.s006.tif]

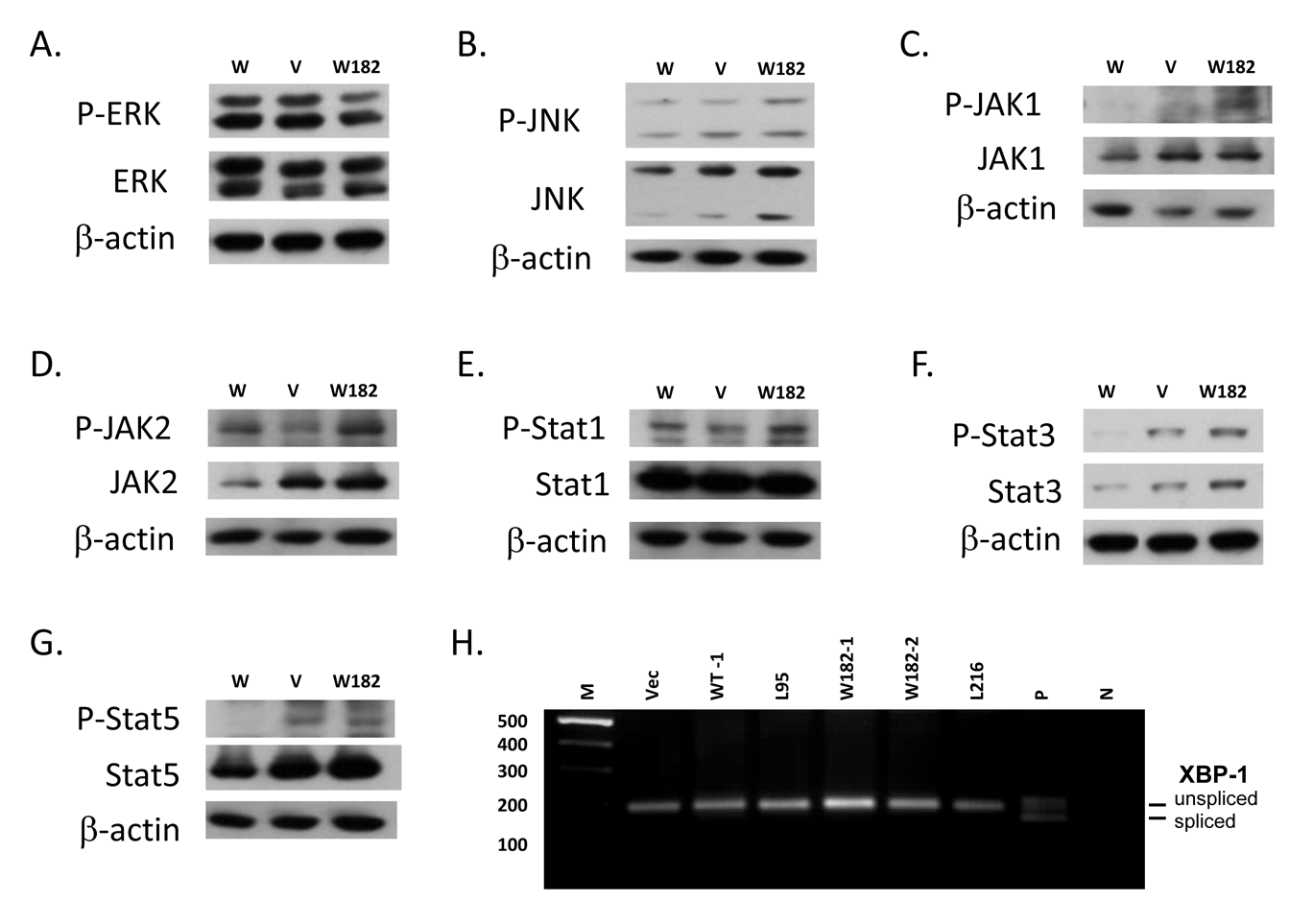

Supplement: Figure S7 — Phosphorylation profile of signaling molecules in 3 stable clones: Wt, vector only, and W182 were evaluated for 7 proteins: (A) ERK. (B) JNK. (C) JAK1. (D) JAK2. (E) Stat1. (F) Stat3. (G) Stat5. W182 mutants showed higher phosphorylation level than MOCK and Wt in most of the proteins except ERK and Stat 1. (H) Assessment for endoplasmic reticulum (ER) stress showed no splicing of mouse X box binding protein-1 (XBP-1) for all 3 HBV S truncation mutants (L95, L216, W182), and the Wt, which meant negative for ER stress. (TIF) [file pone.0089753.s007.tif]
