# Supplementary material for: Identification of Transforming Hepatitis B Virus S Gene Nonsense Mutations Derived from Freely Replicative Viruses in Hepatocellular Carcinoma
Source: PLoS One. 2014 Feb 24;9(2):e89753. doi: 10.1371/journal.pone.0089753 (PMC3933656; doi:10.1371/journal.pone.0089753)
Supplement: Table S1 — Nonsense mutations of Hepatitis B virus S gene in 50 HBV-related HCC patients. (DOCX) [file pone.0089753.s008.docx]

**Table S1. Nonsense mutations of Hepatitis B virus S gene in 50 HBV-related HCC patients**

| **No.** | **A.A. sequence** | **genotype** | **T** | **N** | **Note** |
| --- | --- | --- | --- | --- | --- |
| **1** | **S61Stop** | **C** | **1** | **1** | **This tumor had mutations in both T and N** |
| **2** | **C69Stop** | **C** | **2** | **0** | **Both tumors also had W182Stop** |
| **3** | **L95Stop** | **B** | **1** | **0** |  |
| **4** | **W182Stop** | **C** | **6** | **2** | **2 tumors had mutations in both T and N** |
| **5** | **L216Stop** | **C** | **2** | **0** | **One tumor also had W182Stop** |
| ***Total*** |  |  | ***12*** | ***3*** |  |

HBV, hepatitis B virus; HCC, hepatocellular carcinoma; A.A., amino acid; T, tumor tissue; N, paired non-tumor liver tissue.
