# Supplementary material for: Identification of Transforming Hepatitis B Virus S Gene Nonsense Mutations Derived from Freely Replicative Viruses in Hepatocellular Carcinoma
Source: PLoS One. 2014 Feb 24;9(2):e89753. doi: 10.1371/journal.pone.0089753 (PMC3933656; doi:10.1371/journal.pone.0089753)
Supplement: Table S2 — Clinical Characteristics of the 25 HBcAg(+) HCC and 25 matched HBcAg(+) HCC patients. (DOCX) [file pone.0089753.s009.docx]

**Table S2. Clinical Characteristics of the 25 HBcAg(+) HCC and 25 matched HBcAg(+) HCC patients**

| **Clinical variables** | **HBcAG(+) HCC** | **HBcAg (-) HCC** | ***P* value**^a^ |
| --- | --- | --- | --- |
| **Patient No.** | 25 | 25 |  |
| **Gender** |  |  |  |
| **Male** | 20 | 20 | 1.0000 |
| **Female** | 5 | 5 |  |
| **Age (year)** | 51.8 | 51.4 | 0.568^b^ |
| **(range)** | 31-74 | 27-71 |  |
| **Cirrhosis** |  |  |  |
| **(+)** | 23 | 12 | **<0.001** |
| **(-)** | 2 | 13 |  |
| **HCC grading**^c^ |  |  |  |
| **I** | 1 | 0 | **combined as a reference group** |
| **II** | 9 | 4 |  |
| **III** | 14 | 17 | 0.227 |
| **IV** | 1 | 4 | 0.145 |
| **Tumor size** |  |  |  |
| **≦2 cm** | 9 | 2 | **0.039** |
| **＞2 cm** | 16 | 23 |  |
| **Pathology Stage**^d^ |  |  |  |
| **I** | 14 | 9 | **Reference group** |
| **II** | 9 | 7 | 0.242 |
| **III** | 2 | 8 | 0.125 |
| **IV** | 0 | 1 | 1.000 |
| **Disease Free Survival**^e^ |  |  |  |
| **Recurred / censored** | 20/2 | 19/4 | 0.686 |
| **Median survival (95%CI)** | 21.2 months (12.2, 50.2) | 9.8 months (6.8, 35.7) |  |
| **Overall Survival** |  |  | 0.793 |
| **Death/censored** | 15/10 | 18/7 |  |
| **Median survival (95%CI)** | 72.2 months (26.1, -- | 44.0 months (22.5, 102.6) |  |

^a^ Exact test for association with matched pair data, except for the variable ‘age’.

^b^ For age, paired t-test is used.

^c^ Edmondson-Steiner grading

^d^ According to the American Joint Committee on cancer (AJCC) staging system, 6^th^ edition.

^e^Five patients had no disease free survival data, since the recurrent date were uncertain, but all died of tumor.
