# Supplementary material for: Identification of Transforming Hepatitis B Virus S Gene Nonsense Mutations Derived from Freely Replicative Viruses in Hepatocellular Carcinoma
Source: PLoS One. 2014 Feb 24;9(2):e89753. doi: 10.1371/journal.pone.0089753 (PMC3933656; doi:10.1371/journal.pone.0089753)
Supplement: Table S3 — Primer sets for detection of the full-length Hepatitis B Virus DNA genome. (DOCX) [file pone.0089753.s010.docx]

**Table S3. Primer sets for detection of the full-length Hepatitis B Virus DNA genome**

| **Genes** | **Primers** | |
| --- | --- | --- |
|  | **Forward** | **Reverse** |
| **PreCore/Core** | **GCC TTC TCA TCT ACC GGA CC** | **GTA TGG TGA GGT GAA CAA TG** |
|  | **TCT GTA TCG AGA AGC CTT AG** | **AGT TTC CCA CCT TAT GAG TC** |
| **X** | **GAT CCA TAC TGC TGC GGA ACT CC** | **GTT CAC GGT GGT CTC CAT G** |
|  | **TCT GTG CCT TCT CAT CTG C** | **AGC TTG GAG GCT TGA ACA G** |
| **Polymerase** | **GGTTTCACATTTCCTGTCTTAC** | **GCAGGGTCCAACTGATGATC** |
|  | **GTCTGTACAACATCTTGAGTC** | **CAGGATCCAGTTGGCAGCAC** |
| **PreS/S** | **GAGAGTCCACACGTAGCG** | **GACTCTGTGGTATTGTGAGG** |
|  | **CTCACAACTGTGCCAGCAGC** | **CTGAGGATGAGTGTCCCTTAG** |
|  | **GGACCCTGTACCGAACATG** | **GTTCCTGTGGCAATGTGCC** |
|  | **CTCCAGTCACTCACCAACC** | **CCTTGATAGTCCAGAAGAACC** |
